# The impact of musculoskeletal injuries sustained in road traffic crashes on work-related outcomes: a protocol for a systematic review

**DOI:** 10.1186/s13643-018-0869-4

**Published:** 2018-11-20

**Authors:** Elise M. Gane, Charlotte L. Brakenridge, Esther J. Smits, Venerina Johnston

**Affiliations:** RECOVER Injury Research Centre, The University of Queensland, Oral Health Centre, Herston, QLD 4006 Australia

**Keywords:** Return to work, Work, Employment, Work performance, Traffic accidents, Musculoskeletal diseases, Wounds and injuries, Whiplash injuries, Pain

## Abstract

**Background:**

Musculoskeletal injuries (strains/sprains, contusions, dislocations, and fractures) are the most common injury sustained in a road traffic crash. They can have a long-term impact upon the ability to engage in work. Persisting symptoms as well as poor physical and psychological recovery may reduce the ability to return to or remain at work necessitating the use of sick leave or alternate duties to enable a gradual return to full duties. There is also a need to investigate rates of return to work, along with other work-related outcomes in this population so that interventions designed to facilitate return to work can be targeted to this clinical population. In addition, there is a need to explore factors associated with work-related outcomes in people with musculoskeletal injuries subsequent to a road traffic crash.

**Methods:**

A systematic review will be conducted to determine the impact of sustaining a musculoskeletal injury during a road traffic crash on an individual’s ability to work. Observational studies will be identified by searching six electronic databases for reports of adults having sustained musculoskeletal injuries during a road traffic crash. Studies featuring paediatric cohorts or those with neurological injuries will be excluded. To be eligible for inclusion, studies must report at least one of the following work-related outcomes: return to work status/rate, sick leave, work ability, work capacity, and health-related work productivity loss. The methodological quality of included studies will be assessed with the National Institutes of Health National Heart, Lung, and Blood Institute Study Quality Assessment Tools for observational cohort and cross-sectional studies, and case-control studies.

**Discussion:**

The results of this systematic review may increase our knowledge of work-related outcomes and understanding of the associated factors for people with musculoskeletal injuries following road traffic crashes. Future studies could use the results to plan interventions and influence policy and legislation, and raise awareness of the needs of this clinical population.

**Systematic review registration:**

Registered on PROSPERO, reference number CRD42018099252, dated 14 August 2018.

**Electronic supplementary material:**

The online version of this article (10.1186/s13643-018-0869-4) contains supplementary material, which is available to authorized users.

## Background

Road traffic crashes and their associated mortality and morbidity are a recognised global health problem [1, 2]. Road injury was the tenth leading cause of death worldwide in 2015, with 1.3 million fatalities (2.4% of global deaths) [3]. The WHO predicts the number of fatalities will increase to position road injury as the seventh leading cause of death globally by 2030 [4]. In addition to the loss of life, road traffic crashes can cause significant disease burden for those who survive. Road injury had the sixth highest disease burden in 2015, ahead of diabetes and human immunodeficiency virus/acquired immunodeficiency syndrome [5]. There is also an associated economic burden for individual countries, with the average cost of road traffic deaths and injuries being 3% of gross domestic product [6].

Non-fatal injuries sustained in road traffic crashes can range from minor to catastrophic, and include a variety of injuries from musculoskeletal injury [7] to traumatic brain injury [8] and spinal cord injury [9]. Musculoskeletal injuries affect joints, bones, muscles, and the spine [10]. These injuries are the most common sustained during road traffic crashes and constitute the largest proportion of compensation claims submitted [7]. Symptoms such as pain can also persist and manifest themselves into chronic conditions [11] such as whiplash-associated disorder (WAD); and individuals can experience disability and reduced health-related quality of life [12, 13]. Participation in activities of daily living, fulfilling social and familial roles and engaging in paid employment could become difficult. In particular, being gainfully employed has flow-on effects for the financial security of families and society as a whole, as well as being a source of self-identity and self-esteem for the individual [14]. There is an existing knowledge base of the impact of brain injury [15], spinal cord injury [16, 17], and psychological sequelae [18] on work-related outcomes following road traffic crash. There has not yet been a systematic review of the impact of musculoskeletal injuries sustained in road traffic crashes on work-related outcomes.

It is well-recognised that employment is a determinant of health, and equally, that poor health is a major contributor to loss of work capacity and unemployment [19, 20]. Extended absence from work is associated with harm to mental and physical health [19, 20]. There is also a growing body of evidence that re-engagement in work after a period of illness or injury can promote recovery from that illness or injury [21], and a growing movement of support for the health benefits of ‘good work’ [19, 22]. The WHO implemented a global plan of action on workers’ health during 2008–2017, particularly targeting improvements to health coverage for workers in small companies, informal work, and the agriculture sector [23, 24]. Health coverage is necessary to meet the demands of the cost of work-related health problems, which can result in a 4–6% loss of gross domestic product (GDP) for the majority of countries [24]. During the 2015–2016 financial year in Australia, a total of AU$37.2 billion was spent on income support for individuals unable to work due to poor health, the majority of which (AU$18.7 billion) were employer-provided entitlements [25]. This is evidence of a vested interest from employers in the rehabilitation and management of workers with poor health. To address this issue, some employers have adopted the use of rehabilitation interventions delivered in the workplace, which have been shown to reduce the time between injury and first attempt at return to work, as well as reducing the cumulative duration of sickness absence [26]. It is clearly in the interests of the injured person as well as their employer to facilitate early return to work, provide workplace-based interventions, and access to income support.

There is a need to investigate rates of return to work in people with musculoskeletal injuries following road traffic crash so that interventions designed to facilitate return to work can be targeted to this clinical population. In addition to return to work rates, it is also important to explore other work-related outcomes such as the way in which an injured person returns to work (i.e. with a graduated program of work hours and duties) or the absolute number of those who have or have not re-entered the workforce. Alternative duties may be prescribed on the basis of a work capacity assessment, which is an objective assessment of a worker’s functional capacity. Work capacity also contributes to work ability, which is a more holistic concept that encompasses the physical and mental ability of a person to meet the demands of their job—a balance between a person’s resources and their work demands, determined by work capacity as well as knowledge, skills, values, attitudes, motivation, and the work itself [27]. Some injured persons might access sick leave (paid leave provided by the employer) before or during their return to work that can be measured in monetary terms. Being absent from work due to a health condition and therefore not being productive at the workplace is referred to as absenteeism [28]. If an injured person is at work, but working at a reduced performance level, this is called presenteeism; presenteeism can also be referred to as work performance [28, 29]. The evaluation of absenteeism, presenteeism, and employee wages combined produces a measure of health-related work productivity loss [30, 31]. Clearly describing return to work rates, how a person returns to work, work capacity and ability, sick leave, and presenteeism after road traffic-related musculoskeletal injuries will assist policy makers and employers to assess the cost-effectiveness of funding services that assist injured workers back to work.

### Research aim/objectives

The primary aim of this systematic review is to determine the impact of sustaining a musculoskeletal injury during a road traffic crash on five work-related outcomes: the rate of return to work following injury, the utilisation of sick leave, work capacity, work ability, and health-related work productivity loss. The secondary aim is to determine factors associated with these work-related outcomes in people with musculoskeletal injuries as a result of a road traffic crash.

### Study rationale

The existing evidence on work and work-related outcomes as a consequence of musculoskeletal injuries sustained in road traffic crashes has not yet been synthesised with a systematic review. This synthesis is required to inform future studies in the field of promoting return to work after these injuries, to inform policy and legislation, and to raise awareness of the long-term consequences of injuries that can be minor in nature, but significant in their effect on the lives of individuals in the community.

## Methods

### Protocol preparation and registration

This systematic review protocol has been drafted in line with the Preferred Reporting Items for Systematic review and Meta-Analysis Protocols (PRISMA-P) statement [32] [see Additional file 1]. Registration of this systematic review can be viewed within the International prospective register of systematic reviews (PROSPERO) database (CRD42018099252). The final systematic review will be presented in compliance with the PRISMA statement [33]. If there are sufficient studies with which to perform a meta-analysis, the review will also be presented in compliance with the Meta-analysis Of Observational Studies in Epidemiology (MOOSE) statement [34].

### Inclusion criteria for considering studies for this review

Studies with an observational design including cross-sectional, prospective cohort, retrospective cohort, and case-control will be accepted. Randomised controlled trials of interventions and other intervention designs will be excluded because the process of natural recovery is the focus of this review, not the effect of a specific and deliberate intervention aimed at changing the process of natural recovery. Case studies will also be excluded. Participants must be adults who have sustained a musculoskeletal injury during a road traffic crash. Studies investigating neurological injuries such as spinal cord injury and traumatic brain injury, or any injury sustained by a paediatric cohort (age < 18 years) will be excluded. If studies include participants with either musculoskeletal or neurological injuries (but not both), they will be included if results are reported separately for those with musculoskeletal injuries. Fault status or compensation status of participants will not be used to determine the eligibility of a study, but this information will be recorded and used to assist the interpretation of results. Fault status describes whether or not an individual involved in a road traffic crash was responsible for the crash (‘at fault’ or ‘not at fault’).

Eligible studies will also be required to report their findings using at least one of the following work-related outcomes: (i) return to work status/rate, (ii) sick leave, (iii) work ability, (iv) work capacity, and (v) health-related work productivity loss. Examples of specific outcome measures used to measure these five work-related outcomes are presented in Table 1.

**Table 1:** To be eligible for inclusion in this review, studies need to report on at least one of the work-related outcomes listed here. Studies may choose to report non-standardised outcome measures for work ability, work capacity, and health-related productivity loss. Such studies will not be excluded from this review

Work-related outcome	Example of how outcome might be measured
Return to work status/rate	Percentage of participants who have returned to work by a certain time following injury. The type of work (i.e. reduced hours, modified duties) may also be reported.
Sick leave	Use of sick leave (e.g. yes or no, number of hours/days) Cost of sick leave (e.g. wages)
Work ability	Work Ability Index [44]
Work capacity	Functional Capacity Evaluation [45]
Health-related work productivity loss	Stanford Presenteeism Scale [46] WHO’s Health and Work Performance Questionnaire [29] Lam Employment Absence and Productivity Scale [47]

### Search method for identification of studies

Six electronic databases will be searched for relevant studies for this review. The search strategy will be executed by one author (EG) within the following electronic databases: PubMed, EMBASE, CINAHL, Web of Science, PsycINFO, and Australian Transport Index (grey literature source). The search strategy will be a combination of search strings that cover the areas of (1) work/employment, (2) road traffic crash, (3) observational study design, and (4) musculoskeletal injuries. Search strings will first be built within PubMed and modified accordingly for the other databases. The PubMed search strategy is presented in Table 2. Consistency across databases will be maintained where possible. Terms will be searched within multiple fields where available (e.g. MeSH or Emtree, keyword, title, abstract). Fields chosen will be dependent upon the database in question. The expertise of a university librarian will be utilised in the drafting of the search strategies. There will be no date limit applied to the search; however, search results will be limited to the English language as there is no funding available for the translation of articles. If relevant systematic reviews are captured by the search, their reference lists will be reviewed for eligible studies. When the final list of included studies has been determined, their reference lists will also be reviewed for relevant studies, and each study will be entered into Web of Science to determine other potentially relevant papers that have cited an included study (forward citation searching). The authors’ own personal reference libraries will also be checked for additional papers, including non-published abstracts from conferences and professional societies. If relevant abstracts are identified, contact will be made with the authors for further information.

**Table 2:** The PubMed search strategy

Work related search terms	
#1	work [Title/abstract] OR job [Title/abstract] OR jobs [Title/abstract] OR occupation [Title/abstract] OR occupations [Title/abstract] OR occupational [Title/abstract] OR vocation [Title/abstract] OR vocations [Title/abstract] OR vocational [Title/abstract] OR employee [Title/abstract] OR employees [Title/abstract] OR employer [Title/abstract] OR employers [Title/abstract] OR worker [Title/abstract] OR workers [Title/abstract] OR workplace [Title/abstract] OR employability [Title/abstract] OR unemployment [Title/abstract] OR employment [Title/abstract] OR absentee [Title/abstract] OR “sick listed”[Title/abstract] OR sicklisted [Title/abstract] OR “sick leave”[Title/abstract] OR “sick absence”[Title/abstract] OR “sickness leave”[Title/abstract] OR “sickness absence”[Title/abstract] OR “sick days”[Title/abstract] OR “sick day”[Title/abstract] OR “illness day”[Title/abstract] OR “illness days”[Title/abstract] OR absenteeism [Title/abstract] OR presenteeism [Title/abstract] OR “workday loss”[Title/abstract] OR “workdays lost”[Title/abstract] OR workloss [Title/abstract] OR “disability evaluation”[Title/abstract] OR “disability prevention”[Title/abstract] OR “disability leave”[Title/abstract] OR “functional capacity evaluation”[Title/abstract] OR productivity [Title/abstract]
#2	work [MeSH] OR employment [MeSH] OR “sick leave”[MeSH] OR “work capacity evaluation”[MeSH]
#3	1 OR 2
Road traffic crash-related search terms	
#4	(Car [Title/abstract] OR Cars [Title/abstract] OR Truck [Title/abstract] OR Trucks [Title/abstract] OR Automobile [Title/abstract] OR Automobiles [Title/abstract] OR Vehicle [Title/abstract] OR Vehicles [Title/abstract] OR Vehicular [Title/abstract] OR Cycle [Title/abstract] OR Cycles [Title/abstract] OR Cyclist [Title/abstract] OR Cyclists [Title/abstract] OR Cycling [Title/abstract] OR Bicycle [Title/abstract] OR bicycles [Title/abstract] OR Pedestrian [Title/abstract] OR Pedestrians [Title/abstract] OR Passenger [Title/abstract] OR Passengers [Title/abstract] OR Driver [Title/abstract] OR Drivers [Title/abstract] OR motor [Title/abstract] OR motorbike [Title/abstract] OR motorbikes [Title/abstract] OR motorbiker [Title/abstract] OR motorbikers [Title/abstract] OR motorcar [Title/abstract] OR motorcars [Title/abstract] OR motorcycle [Title/abstract] OR motorcycles [Title/abstract] OR motorcycling [Title/abstract] OR motorcyclist [Title/abstract] OR motorcyclists [Title/abstract] OR motorhome [Title/abstract] OR motorhomes [Title/abstract] OR motorist [Title/abstract] OR motorists [Title/abstract] OR motorvehicle [Title/abstract] OR motorvehicles [Title/abstract] OR transport [Title/abstract] OR transportation [Title/abstract] OR traffic [Title/abstract] OR road [Title/abstract] OR roads [Title/abstract] OR roadside [Title/abstract] OR roadsides [Title/abstract]) AND (accident [Title/abstract] OR accidents [Title/abstract] OR collision [Title/abstract] OR collisions [Title/abstract] OR crash [Title/abstract] OR crashes [Title/abstract] OR crashed [Title/abstract] OR smash [Title/abstract] OR smashes [Title/abstract] OR smashed [Title/abstract])
#5	“Accidents, Traffic”[MeSH]
#6	“Whiplash injuries”[MeSH] OR whiplash [Title/abstract]
#7	(road [Title/abstract] OR traffic [Title/abstract]) AND (injury [Title/abstract] OR injuries [Title/abstract] OR trauma [Title/abstract])
#8	4 OR 5 OR 6 OR 7
Study type	
#9	Crosssectional [Title/abstract] OR cross-sectional [Title/abstract] OR “cross sectional”[Title/abstract] OR observational [Title/abstract] OR casecontrol [Title/abstract] OR case-control [Title/abstract] OR “case control”[Title/abstract] OR cohort [Title/abstract] OR longitudinal [Title/abstract] OR ((prospective [Title/abstract] OR retrospective [Title/abstract]) AND (cohort [Title/abstract] OR study [Title/abstract] OR observational [Title/abstract] OR longitudinal [Title/abstract]))
Musculoskeletal injury	
#10	Musculoskeletal [Title/abstract] OR myofascial [Title/abstract] OR arthralgia [Title/abstract] OR arthropathy [Title/abstract] OR arthritis [Title/abstract] OR arthritic [Title/abstract] OR myalgia [Title/abstract] OR backache [Title/abstract] OR whiplash [Title/abstract]
#11	“Musculoskeletal diseases”[MeSH] OR “Musculoskeletal system”[MeSH] OR “Wounds and injuries”[MeSH] OR “Wounds, Nonpenetrating”[MeSH] OR “Musculoskeletal pain”[MeSH] OR “Back pain”[MeSH] OR “Myofascial pain syndromes”[MeSH] OR Arthralgia [MeSH] OR “Neck pain”[MeSH] OR “brachial plexus neuropathies”[MeSH] OR “Fractures, Bone”[MeSH] OR “Joint Dislocations”[MeSH] OR “Soft tissue injuries”[MeSH] OR “Multiple trauma”[MeSH] OR Orthopedics [MeSH] OR “Orthopedic Procedures”[MeSH]
#12	(Fracture [Title/abstract] OR Fractures [Title/abstract] OR Fractured [Title/abstract] OR sprain [Title/abstract] OR sprains [Title/abstract] sprained [Title/abstract] OR dislocation [Title/abstract] OR dislocations [Title/abstract] OR dislocated [Title/abstract] OR injury [Title/abstract] OR injuries [Title/abstract] OR injured [Title/abstract] OR contusion [Title/abstract] OR contusions [Title/abstract] OR oedema [Title/abstract] OR edema [Title/abstract] OR trauma [Title/abstract] OR multitrauma [Title/abstract] OR multi-trauma [Title/abstract] OR “multi trauma”[Title/abstract] OR orthopaedic [Title/abstract] OR orthopaedics [Title/abstract] OR orthopedic [Title/abstract] OR orthopedics [Title/abstract] OR surgery [Title/abstract] OR ache [Title/abstract] OR pain [Title/abstract]) AND (Arm [Title/abstract] OR arms [Title/abstract] OR “upper limb”[Title/abstract] OR “upper limbs”[Title/abstract] OR “upper extremity”[Title/abstract] OR “upper extremities”[Title/abstract] OR leg [Title/abstract] OR legs [Title/abstract] OR “lower limb”[Title/abstract] OR “lower limbs”[Title/abstract] OR “lower extremity”[Title/abstract] OR “lower extremities”[Title/abstract] OR shoulder [Title/abstract] OR shoulders [Title/abstract] OR “rotator cuff”[Title/abstract] OR humerus [Title/abstract] OR humeral [Title/abstract] OR elbow [Title/abstract] OR elbows [Title/abstract] OR forearm [Title/abstract] OR forearms [Title/abstract] OR radius [Title/abstract] OR radial [Title/abstract] OR ulna [Title/abstract] OR ulnar [Title/abstract] OR wrist [Title/abstract] OR wrists [Title/abstract] OR hand [Title/abstract] OR hands [Title/abstract] OR finger [Title/abstract] OR fingers [Title/abstract] OR thumb [Title/abstract] OR thumbs [Title/abstract] OR spine [Title/abstract] OR spinal [Title/abstract] OR cervical [Title/abstract] OR thoracic [Title/abstract] OR thorax [Title/abstract] OR lumbar [Title/abstract] OR sacral [Title/abstract] OR sacrum [Title/abstract] OR neck [Title/abstract] OR chest [Title/abstract] OR back [Title/abstract] OR pelvis [Title/abstract] OR pelvic [Title/abstract] OR hip [Title/abstract] OR hips [Title/abstract] OR knee [Title/abstract] OR knees [Title/abstract] OR ankle [Title/abstract] OR ankles [Title/abstract] OR thigh [Title/abstract] OR thighs [Title/abstract] OR femur [Title/abstract] OR femoral [Title/abstract] OR tibia [Title/abstract] OR tibial [Title/abstract] OR fibula [Title/abstract] OR fibular [Title/abstract] OR shin [Title/abstract] OR shins [Title/abstract] OR foot [Title/abstract] OR feet [Title/abstract] OR toe [Title/abstract] OR toes [Title/abstract] OR metatarsal [Title/abstract] OR metatarsals [Title/abstract] OR mandibular [Title/abstract] OR maxillofacial [Title/abstract] OR ligament [Title/abstract] OR ligaments [Title/abstract] OR muscle [Title/abstract] OR muscles [Title/abstract] OR “soft tissue” [Title/abstract] OR tendon [Title/abstract] OR tendons [Title/abstract])
#13	10 OR 11 OR 12
Combined	
#14	3 AND 8 AND 9 AND 13
#15	Limit to English

### Data collection and analysis

The primary author (EG) conducting the database search will export the search findings into the referencing software Endnote (Version ≥X7, Clarivate Analytics) and remove duplicate entries from the list of results. Following this, titles and abstracts will be screened independently by two authors (EG, CB). Each full text will be sourced (including via direct communication with authors if necessary) and independently reviewed by two authors (EG and either CB or ES). The resolution of any discrepancies will occur with the input of the senior author (VJ). All authors will then agree upon the final list of included studies.

Data extraction from and methodological quality assessment of the included studies will be conducted by two authors independent of each other (EG and either CB or ES), with any disputes resolved by the senior author (VJ). The data to be extracted from each included study is listed in Table 3. The main outcomes extracted from each study will be one or more of the following: (i) return to work status/rate, (ii) sick leave, (iii) work ability, (iv) work capacity, and (v) health-related work productivity loss. Factors associated with these work-related outcomes will depend upon the covariates used in individual studies. Common covariates expected to be investigated include age, sex, compensation status, and injury severity. The data will be managed within Microsoft Excel (Version 2016, Microsoft). The methodological quality of included studies will be assessed with the National Institutes of Health (NIH) National Heart, Lung, and Blood Institute (NHLBI) Study Quality Assessment Tools for observational cohort and cross-sectional studies, and case-control studies (two tools) [35]. The NHI NHLBI tools are 12 to 14 items in length and have been used in previous systematic reviews of observational studies [36–38]. Each item is proposed as a question, and the possible answers are yes, no, or other (cannot determine, not applicable, or not reported). The NHI NHLBI tools are accompanied by instructions to enable authors to score appropriately. After scoring, authors will weigh the evidence and judge if a study can be rated as ‘good’, ‘fair’, or ‘poor’ overall based on instructions given by the NHI NHLBI tools. Good studies have the least risk of bias, and results are considered valid. Fair studies have some risk of bias that does not reduce the validity of the presented results. Poor studies have high risk of bias and should be excluded from the results of a review, except in circumstances where no other evidence is available. The overall quality of the evidence will be rated independently by two authors (EG and either CB or ES) using the Grading of Recommendations Assessment, Development and Evaluation (GRADE) system [39–41].

**Table 3:** Data to be extracted from included studies

Citation	Title of article Lead author Year published Journal
Study Aim	Aim/hypothesis/objective
Methods	Study design Study setting (including jurisdiction) Participants—inclusion/exclusion criteria Method of recruitment Informed consent
Outcome measures	All outcome measures used, any listed psychometrics and associated references reported in the paper Assessment time points
Results	Participation rate Similarity between participants vs non-participants, and those who remain vs lost to follow up Demographics of included cohort Results for outcome measures of interest to this review ((i) return to work rate, (ii) sick leave, (iii) work ability, (iv) work capacity, and (v) health-related work productivity loss) Covariates or confounders reported as significantly or not significantly related to the outcome measures of interest
Discussion	Author listed limitations
Conclusion	Concluding message

### Data synthesis and reporting

Level of agreement on the decision to include or exclude a study will be recorded between authors for the title/abstract and full-text screening stages of the review. Key study characteristics (e.g. sample size, participant demographics) and primary and secondary outcomes will be presented in table format. Regarding Aim 1, effect sizes (with variability estimates) for each primary work outcome will be reported in the table. A narrative synthesis of results will then be presented by the work-related outcome of interest (return to work status/rate, sick leave, work ability, work capacity, and health-related work productivity loss), with reference to the quality of the studies (NHI NHLBI rating of good, fair, or poor). A meta-analysis will be conducted for each work outcome provided there are at least two studies reporting on this outcome (with variability estimates) to make meta-analysis possible. Continuous outcomes (e.g. days of sick leave and work ability score) will be combined into a pooled mean and sampling variance. Categorical outcomes (e.g. percentage of people returned to work) will be transformed using the Freeman-Tukey double arcsine method [42] in order to calculate an overall percentage from a set of percentages.

Regarding Aim 2, a narrative synthesis of factors associated with work outcomes will also be presented. Providing that at least two studies report the same factor in relation to the same outcome, a random effects meta-analysis will be used to pool the effect of each factor to best account for heterogeneity.

Meta-analyses will be conducted in Comprehensive Meta-Analysis (v3.0, Biostat, USA). Heterogeneity will be tested using Cochran’s *Q* test [43]. Sensitivity analyses will be conducted to exclude the studies rated as ‘poor’. If there are sufficient studies, sub-group analyses will occur to explore the effect of gender, injury type, compensation status, follow-up duration, and self-reported vs objectively reported data on any of the outcomes appropriate for meta-analysis.

## Discussion

This systematic review has the potential to raise awareness of the impact of musculoskeletal injuries on employment rates and work capacity as well as the cost of supporting injured persons in their efforts to return to the workforce after a road traffic crash. Furthermore, this systematic review may identify factors associated with successfully (or unsuccessfully) returning to work that could be trialled in future studies of early screening tools to identify those at risk of poor recovery.

In the preparation of findings within this review, compensation status and local legislative requirements will be taken into account as both are likely to be directly related to individual outcomes. For example, not all jurisdictions in Australia fund wage replacement for those who are unable to work as a result of injuries sustained in a road traffic crash. These terms are normally dictated by local legislation. In addition, employers are not obliged to provide modified duties or other concessions to these individuals in some jurisdictions. In order to argue for compassionate change to legislation, a better understanding of the impact of a musculoskeletal injury sustained in a road traffic crash on an individual’s ability to return and remain at work is needed.

## Conclusion

This systematic review will help inform future interventions to promote return to work following crash-related musculoskeletal injury. This review has the potential to inform policy and legislation, particularly in relation to income support for individuals with traffic-related injuries. Finally, this review will describe the potential for seemingly minor musculoskeletal injuries to have long-term implications for individuals as well as their communities at large.

## Additional file

Additional file 1: PRISMA P. (DOCX 30 kb)

## Abbreviations

GRADE: Grading of Recommendations Assessment, Development and Evaluation
MOOSE: Meta-analysis Of Observational Studies in Epidemiology
NHLBI: National Heart, Lung, and Blood Institute
NIH: National Institutes of Health
PRISMA-P: Preferred Reporting Items for Systematic review and Meta-Analysis Protocols
PROSPERO: International prospective register of systematic reviews
Qld: Queensland
WAD: Whiplash-associated disorder
WHO: World Health Organization
